# Linearly Responsive, Reliable, and Stretchable Strain Sensors Based on Polyaniline Composite Hydrogels

**DOI:** 10.3390/gels11120966

**Published:** 2025-11-29

**Authors:** Chubin He, Xiuru Xu

**Affiliations:** State Key Laboratory of Radio Frequency Heterogeneous Integration, College of Physics and Optoelectronic Engineering, Shenzhen University, Shenzhen 518060, China

**Keywords:** piezoresistive strain sensors, conductive hydrogel, polyaniline, linearly response

## Abstract

Conductive hydrogels are ideal for flexible strain sensors, yet their practical use is often limited by water evaporation, signal hysteresis, and structural instability, which impair linearity, durability, and long-term reliability. To overcome these challenges, we developed a robust multiple-network hydrogel composed of poly(vinyl alcohol) (PVA), polyacrylic acid (PAA), in situ polymerized polyaniline (PANi), and the ionic liquid [EMIM][TFSI]. The resulting composite exhibits an exceptional linear piezoresistive response across its entire working range—from rest to fracture strain of 290%—together with high conductivity (0.68 S/cm), fast response/recovery (0.34 s/0.35 s), and a maximum gauge factor of 2.78. Mechanically robust (tensile strength ≈ 3.7 MPa, modulus ≈ 1.3 MPa), the hydrogel also demonstrates outstanding cyclic durability, withstanding over 12,000 stretching–relaxation cycles, and markedly improved dehydration resistance, retaining about 60% of its mass after 3 days at room temperature. This work provides a holistic material solution for developing high-performance, reliable strain sensors suitable for wearable electronics and soft robotics.

## 1. Introduction

Stretchable conductors are fundamental components for next-generation technologies, including wearable electronics, soft robotics, human health monitors, and human–machine interfaces [1,2,3,4,5]. While conductive elastomers fabricated by blending conductive nanofillers (e.g., carbon nanoparticles [6], carbon nanotubes (CNTs) [7,8], graphene [9,10,11], and metal nanowires [12,13,14]) into polymer matrices have demonstrated promising conductivity, they often suffer from structural and mechanical instability. This is primarily due to the modulus mismatch between rigid conductive fillers and soft elastomers, leading to compromised mechanical performance and unreliable sensing, particularly under large strain [15]. Therefore, developing stretchable conductors that maintain both stable conductivity and mechanical integrity over a wide working strain range represents a critical challenge.

Conductive hydrogels have emerged as a compelling alternative, offering inherent advantages such as high stretchability, softness, and biocompatibility. Among them, polyaniline (PANi)-based hydrogels are particularly attractive for epidermal electronics and soft robots due to the formation of intrinsically conductive and entangled networks [16]. For instance, Wang et al. used a solution-processable approach to develop a PANi/polyacrylic acid/phytic acid composite for sensitive strain sensors based on dynamic hydrogen bonding and electrostatic interactions [16]. Similarly, Gazit et al. prepared an all-organic, conductive hydrogel by combining a protected dipeptide with PANi, which exhibited good conductivity, excellent stretchability, and self-healing properties [17]. However, many PANi-based hydrogel sensors are limited by poor drying resistance and long-term stability for practical applications. Recent efforts to mitigate drying, such as Sun et al.’s strategy of replacing more than 60 wt.% of water with non-volatile solvents [18], achieved improved anti-drying properties but at a significant cost to conductivity (0.248 mS/cm) and mechanical performance. Furthermore, achieving a high linearity in the sensing response remains a common challenge. Typically, the conductive fillers are dispersed within a non-conducting polymer matrix, leading to a stochastic and nonlinear disruption/reformation of conductive pathways upon stretching, which results in a nonlinear response. Consequently, the development of a conductive hydrogel that simultaneously possesses a wide linear operating range, high sensitivity, robust mechanical tolerance, and exceptional durability remains a formidable challenge.

Herein, we report a strategy to overcome these limitations through a multiple elastic network hydrogel composed of poly(vinyl alcohol) (PVA), polyacrylic acid (PAA), in situ polymerized PANi, and the ionic liquid 1-ethyl-3-methylimidazolium bis[(trifluoromethyl)sulfonyl]imide ([EMIM]TFSI). In this design, the PVA and PAA networks act as a synergistic stabilizer, dispersant, and template for the in situ formation of a hierarchical PANi conductive structure. The incorporation of [EMIM]TFSI serves a triple role: as a plasticizer to enhance elongation (by 2-fold), as an anti-drying agent to inhibit water evaporation, and as an electrolyte to drastically boost electrical conductivity (by 17-fold). We propose that good miscibility between PANi and the PVA-PAA network, facilitated by electrostatic interactions and intermolecular hydrogen bonding, along with the addition of [EMIM]TFSI, enables rapid electron transport and stable ion transfer. This synergy forms a stable PANi-[EMIM]TFSI composite conductive network within the PVA-PAA/PANi/[EMIM]TFSI composite hydrogel. The resulting material simultaneously achieves an excellent gauge factor (~2.91), a wide linear sensing range from 0% to 290% strain, good resilience, fast response/recovery times (~0.34 s/~0.35 s), and outstanding durability over 12,000 stretching–relaxation cycles. It is expected that this composite hydrogel has great potential in human health monitoring, soft robotics, and human–machine interfaces.

## 2. Results and Discussion

### 2.1. The Preparation of PVA-PAA/PANi/[EMIM]TFSI Conductive Hydrogels

**Figure 1:**
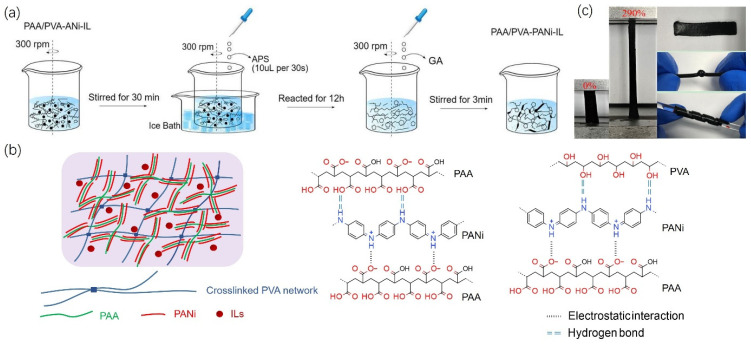
Schematic showing the synthetic process for the PVA-PAA/PANi/[EMIM]TFSI conductive composite hydrogels. (**a**) Fabrication process of the PVA-PAA/PANi/[EMIM]TFSI conductive composite hydrogels, including preparation of the PVA-PAA/PANi/[EMIM]TFSI polymer complex by a one-pot method and formation of the conductive hydrogel by chemical cross-linking. (**b**) The corresponding interactions between polyaniline chains with PVA and PAA. (**c**) A photograph of the PVA-PAA/PANi/[EMIM]TFSI conductive composite hydrogels during relaxed, stretching, twisting, and bending states.

PVA-PAA/PANi/[EMIM]TFSI conductive hydrogels were prepared by two steps, as illustrated in Figure 1a, initiating with the preparation of PVA-PAA/PANi/[EMIM]TFSI polymer complex by a one-pot method, followed by the formation of the conductive hydrogel by chemical cross-linking. For the first step, aniline monomer was mixed with [EMIM]TFSI, PVA, and PAA to form a uniform polymer aqueous solution, and then in situ polymerization was initiated by adding APS as the oxidant, resulting in a homogeneous polymer complex. PAA as proton dopant can effective electrostatic interaction with PANi and the intermolecular hydrogen bonding between the carboxyl group on PAA and the imino groups on PANi (Figure 1b) [16], which is beneficial to stabilize the polyaniline in aqueous solution. With the large-scale physical interactions between PAA and the newly polymerized PANi chains, the hierarchical conductive structures were formed. To restrain the agglomeration of the PANi hierarchical structure and improve the dispersion stability in hydrogel matrixes, PVA polymer chains were added, since the hydroxyl groups on the PVA chains can form hydrogen bonds with the imino groups on PANi chains [19,20].

To modify the conductivity of PANi-based hydrogels, the electronic state of the PANi was adjusted by controlling synthesis conditions. As previously reported, the conductivity of PANi polymer chains was affected by hydrogen ion doping. Due to a large amount of hydrogen ions (most from H_2_SO_4_ and few from the free PAA), we can see that the polymer complex changes from being originally colorless to dark green after being polymerized for 12 h, which indicates that PANi is protonated from its insulating emeraldine base (EB-PANi) to its electrically conductive emeraldine salt (ES-PANi) [21]. Compared with the PAA-PANi sample without H_2_SO_4_, the electrical resistance of the PAA-PANI-H_2_SO_4_ sample was effectively reduced (Figure S3) because the doping of the PANi chain by the sulfate ion increases the electron separation of the PANi chain. Therefore, we selected H_2_SO_4_ and PAA as co-dopants to improve the conductivity of PVA-PAA/PANi/[EMIM]TFSI conductive hydrogels. Simultaneously, [EMIM]TFSI was found to act as an effective plasticizing co-dopant, which may improve the conductivity and maximum tensile strain of PVA-PAA/PANi by a significant amount [22].

Moreover, the mechanical property of conductive hydrogels is one of the most important features for their application, and can be controlled by designing polymer network structures. To achieve high mechanical performance (stretchability, flexibility, and resilience), we designed multiple elastic networks of conductive hydrogels by merging covalent bonds with physical interactions. The PVA-PAA/PANi/[EMIM]TFSI polymer complex was rapidly stirred after the addition of glutaraldehyde (GA), then quickly transferred to an acrylic template and cross-linked at RT for 24 h to form the PVA-PAA/PANi/[EMIM]TFSI conductive hydrogels. For the conductive hydrogel, the aldehyde group in the GA reacted with the hydroxyl group in the PVA to form stable ether bond and develop the chemical cross-link [23]. Meanwhile, there was a large amount of physical cross-linking joints for hydrogen bonding and electrostatic interaction in the cross-linked network (Figure 1b and Figure S2). Therefore, the conductive hydrogel was imparted with excellent flexibility, stretchability, elasticity, and processability. Figure 1c showed the photographs of PVA-PAA/PANi/[EMIM]TFSI conductive hydrogels, which allowed for twisting, bending, and stretching without obvious structural damage or electrical disconnection.

### 2.2. Optimization of Preparation Conditions and Sample Characterization

**Figure 2:**
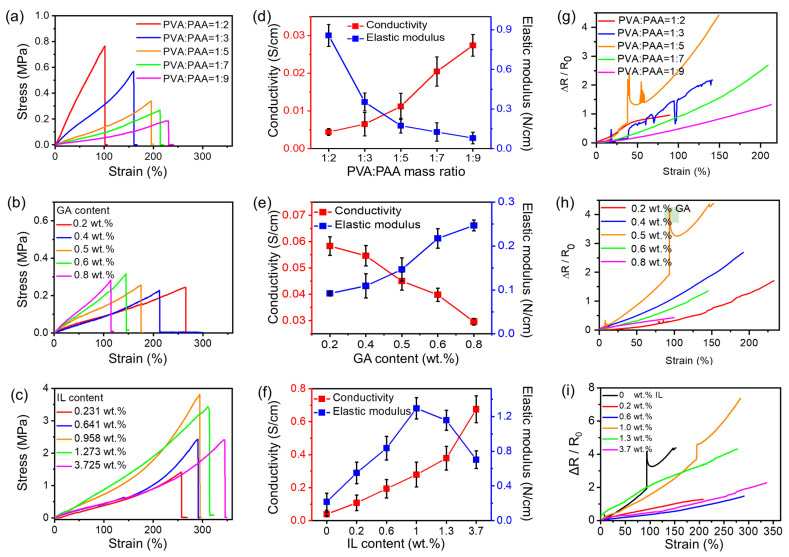
Different PVA-PAA/PANi/[EMIM]TFSI conductive hydrogels can be processed by changing the preparation crafts, like PVA:PAA content, [EMIM]TFSI content, and cross-linker content. The stress–strain behavior of the conductive hydrogels as a function of (**a**) PVA:PAA mass ratios, (**b**) glutaraldehyde (GA) content, and (**c**) [EMIM]TFSI content are shown. The conductivity and tensile modulus of the conductive hydrogels at different (**d**) PVA:PAA mass ratios, (**e**) GA content, and (**f**) [EMIM]TFSI content are also shown. The resistance change rates of the conductive hydrogels at different (**g**) PVA:PAA mass ratios, (**h**) GA content, and (**i**) [EMIM]TFSI content are shown.

In order to build a suitable there-dimensional polymer framework to guide the polymerization of the ANi monomer and better disperse of PANi, we investigate the electronic and mechanical characteristics of PVA-PAA/PANi conductive hydrogels with different PVA:PAA mass ratios and GA weight ratios (showed in Figure 2).

Apparently, higher PVA:PAA mass ratios leads to an increase in stretchability and conductivity and a decrease in tensile strength and tensile modulus at the same time (Figure 2a,d). In addition, as a cross-linking agent, the increase in GA weight ratios reduces the fracture strain and conductivity of the hydrogel and increases their tensile modulus (Figure 2b,e), which is caused by the increased cross-linking degree of the hydrogel network. Meanwhile, we can see from Figure 2g,h that the resistance change rate of the PVA-PAA/PANi conductive hydrogel increases first and then decreases with the increase in strain elongation during the increases in the PVA:PAA mass ratio or GA weight ratios. When PVA:PAA mass ratio = 1:5 and GA weight ratios = 0.48 wt.%, the PVA-PAA/PANi conductive hydrogel showed the largest resistance changing rate. On the other hand, the [EMIM]TFSI ionic liquid simultaneously acted as a plasticizer and conductive agent in the PVA-PAA/PANi/[EMIM]TFSI conductive hydrogel, affecting the electrical and mechanical properties of the conductive hydrogel. Obviously, higher [EMIM]TFSI content leads to increased tensile strain and conductivity (Figure 2c,f). As the [EMIM]TFSI content increases, the mechanical strength and tensile modulus decreases. However, when [EMIM]TFSI content = 0.96 wt.%, the mechanical strength and tensile modulus reach the maximum and then decrease (Figure 2c,f). And at [EMIM]TFSI content = 0.96 wt.%, the resistance change rate of PVA-PAA/PANi/[EMIM]TFSI is also the largest with increasing strain (Figure 2i). Therefore, the PVA-PAA/PANi/[EMIM]TFSI with a [EMIM]TFSI content of 0.958 wt.% shows the best mechanical properties and strain sensitivity. From the above, we deliberate that the PVA-PAA/PANi/[EMIM]TFSI conductive hydrogel containing PVA:PAA mass ratio = 1:5, 0.48 wt.% GA, and 0.96 wt.% [EMIM]TFSI is the optimum choice, which shows excellent stretching capacity (fractured at ~290% strain), superior tensile strength (3.7 MPa), better tensile modulus (~1.3 N/cm), and good electrical conductivity (0.675 S/cm^2^). Those results are better than most stretchable PANi-based conductive hydrogels reported so far [24,25,26].

**Figure 3:**
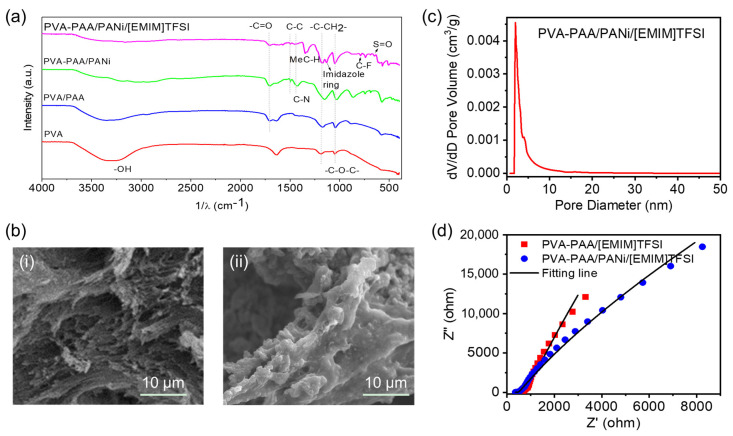
(**a**) FT-IR spectra of different hydrogel samples. (**b**) The SEM of different hydrogel samples without (**i**) and with (**ii**) polyaniline. (**c**) Pore size distribution of the PVA-PAA/PANi/[EMIM]TFSI conductive hydrogels. (**d**) The electrochemical impedance spectroscopy (EIS) of the PVA-PAA/[EMIM]TFSI and PVA-PAA/PANi/[EMIM]TFSI conductive hydrogels.

We also explored the mechanism of the resultant conductive hydrogels by analyzing the microstructure features. The FT-IR spectra was carried out to confirm the structure of the PVA-PAA/PANi/[EMIM]TFSI conductive hydrogel, which is shown in Figure 3a. Bands at 3297, 1188, and 1690 cm^−1^ were assigned to the stretching vibration tensile peaks of free hydroxyl groups, -C-CH_2_- in the polymer backbone and -C=O in the carboxyl group of polyacrylic acid, respectively. And the peak at 1082 cm^−1^ belonged to the C-O-C stretching vibration, indicating that PVA underwent cross-linking of glutaraldehyde to form an ether bond. Peaks at ~1450 and 1340 cm^−1^ were ascribed to the C-C stretching absorption of the quinoid ring in PANi and the C-N stretching vibration in the aromatic ring, which indicated the polymerization of PANi in the PVA-PAA matrix. Peaks at 1338 and 1135 cm^−1^ belonged to the bending stretching vibration of MeC-H connected to the imidazole ring and the imidazole ring skeleton, respectively. The characteristic peaks at ~873 and 686 cm^−1^ were the stretching vibration of the C-F bond and the S=O bond, respectively, indicating that the [EMIM]TFSI ionic liquid was successfully introduced into the PVA-PAA/PANi polymer complex [27,28,29,30]. We also employed Raman spectroscopy to characterize the PVA-PAA/PANi/[EMIM]TFSI conductive hydrogel, aiming to verify the polyaniline structure and its protonation state (Figure S5). The observed characteristic peaks are assigned as follows: the band at 1630 cm^−1^ corresponds to the C=O stretching vibration of benzoquinone; the peak at 1623 cm^−1^ is attributed to the C-C stretching vibration of the benzene ring; a strong band appears at 1569 cm^−1^ (C=C stretching vibration in the quinoid ring). A band near 1490 cm^−1^ is associated with the N-H deformation vibration related to the semiquinone structure. The band at 1356 cm^−1^ provides information on the C~N^+^• vibration of the delocalized polaron structure, featuring a shoulder at 1321 cm^−1^. The benzene ring deformation vibration is linked to the band at 1262 cm^−1^. The band at 1167 cm^−1^ corresponds to the C-H bending vibration of the semiquinoid ring (cation radical segments). The band at 885 cm^−1^ is related to the C-N-C wagging and/or benzene ring deformation in the polarized or bipolaron forms of the emeraldine salt, while the peak at 713 cm^−1^ is associated with the amine deformation reported for the bipolaron form of the emeraldine salt [31,32,33]. To observe the dispersion and structural morphology of the polyaniline/ionic liquid network within the polymer matrix, SEM tests were conducted on hydrogel samples with and without polyaniline (Figure 3b). The sample without polyaniline exhibited a relatively smooth and dense porous structure (Figure 3(bi)). In contrast, the surface of the polyaniline-containing sample was covered with a uniform, dense layer of nanofibrous or particulate polymer (Figure 3(bii)), successfully forming a three-dimensional hierarchical conductive porous network, which is highly beneficial for electron transport and ion diffusion. We also performed a BET test on the PVA-PAA/PANi/[EMIM]TFSI conductive hydrogel and obtained information on the pore size distribution in the conductive hydrogel. The most probable pore size corresponding to the highest peak of the differential curve in Figure 3c was about 2 nm, indicating that there were small mesopores of 2 to 4 nm in the PVA-PAA/PANi/[EMIM]TFSI conductive hydrogel. These small holes provided ion conductive channels, thereby enhancing the conductivity of the PVA-PAA/PANi/[EMIM]TFSI conductive hydrogels. In addition, to further analyze the interactions within the polyaniline/ionic liquid network in the polymer matrix, electrochemical impedance analysis was performed on different gel samples (Figure 3d). The impedance plot of the gel sample containing only the [EMIM]TFSI ionic liquid consists of a semicircular arc in the high-frequency region and a Warburg diffusion line in the low-frequency region. The high-frequency semicircle primarily reflects the ionic conductivity of the gel, specifically the ionic resistance (*R_S_*), while the low-frequency Warburg line represents the ion diffusion process within the gel material, known as the Warburg impedance (*W*). In comparison, the impedance plot of the polyaniline-containing gel sample shows a small semicircular region at high frequencies, followed by a large charge transfer semicircle in the mid-to-high-frequency range, and finally a Warburg diffusion line at low frequencies. The semicircular region appearing in the mid-to-high frequencies typically corresponds to the charge transfer resistance (*R*_c_), describing the ease with which ions (from the electrolyte side) are converted to electrons (on the electrode side) across the interface. The impedance plots of the different gel samples were fitted, and corresponding equivalent circuit models were obtained (Figure S4). The fitting data indicate that the polyaniline-containing gel sample has a smaller *R_S_*, suggesting higher ionic conductivity (Table S1). Additionally, the incorporation of polyaniline effectively reduces the internal electron transport resistance within the gel, thereby lowering the Warburg impedance. This demonstrates the formation of a stable three-dimensional conductive pathway comprising polyaniline and the ionic liquid within the gel network.

### 2.3. The Anti-Drying Property of PVA-PAA/PANi/[EMIM]TFSI Conductive Hydrogels

**Figure 4:**
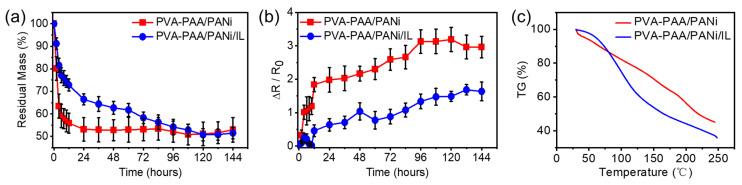
The moisturizing property of the PVA-PAA/PANi/[EMIM]TFSI conductive hydrogel: (**a**) the residual mass for the PVA-PAA/PANi hydrogel and PVA-PAA/PANi/[EMIM]TFSI hydrogel at 25 °C for different times. (**b**) The resistance variation in the PVA-PAA/PANi hydrogel and PVA-PAA/PANi/[EMIM]TFSI hydrogel at 25 °C for different times. (**c**) The thermogravimetric (TGA) curves of PVA-PAA/PANi and PVA-PAA/PANi/IL conductive hydrogels.

The conductive hydrogel samples were stored at room temperature for 6 days to test the anti-drying properties of different PVA-PAA/PANi conductive hydrogels, and the changes in the quality and relative resistance of the conductive hydrogels were recorded over time. The remaining mass of the different conductive hydrogel samples with the storage time is shown in Figure 4a. After leaving them at room temperature for 24 h, the remaining mass of the PVA-PAA/PANi conductive hydrogel decreased to 55%, while the remaining mass of the conductive hydrogel containing [EMIM]TFSI ionic liquid remained around 70%, indicating that the introduction of [EMIM]TFSI ionic liquid can improve the anti-drying performance of the conductive hydrogel. Meanwhile, we also monitored the relative resistance changes in the different conductive hydrogels over time (Figure 4b). With the extension of the storage time, the relative resistance change in the PVA-PAA/PANi conductive hydrogel first had a rapid increase process, and then gradually increased. In contrast, the relative resistance change in the PVA-PAA/PANi/[EMIM]TFSI conductive hydrogel changed slightly at first, and then steadily increased. Moreover, the resistance change value of the PVA-PAA/PANi hydrogel was increased to 400% its original value for 24 h, while the resistance change value of the PVA-PAA/PANi/[EMIM]TFSI hydrogel only increased by 60%. Even after 6 days, the relative resistance change in the PVA-PAA/PANi/[EMIM]TFSI conductive hydrogel increased by only 1.5 times. To further characterize the anti-drying performance, we conducted thermogravimetric analysis (TGA) on different hydrogel samples (Figure 4c). As the temperature increased, the sample without the ionic liquid was the first to lose mass, with its curve declining smoothly and without a distinct plateau. In contrast, the sample containing the ionic liquid initially exhibited a plateau (0–75 °C), followed by an accelerated mass loss, with a second brief plateau appearing around 120 °C. The first plateau corresponded to only a 5% mass loss, likely due to the evaporation of free water in the hydrogel. These results indicate that the incorporation of the ionic liquid effectively enhances the thermal stability and anti-drying properties of the PVA-PAA/PANi/[EMIM]TFSI conductive hydrogel.

The improvement of the moisturizing and anti-drying properties of the hydrogel may be attributed to (1) the formation of hydrogen bonds between PVA, PAA, and water, which plays a certain role in the locking of water molecules in the hydrogel; (2) after the addition of [EMIM]TFSI, the system could occur micro-emulsification, and there is a strong dipole–dipole interaction between the ionic liquid and water, so that some water molecules are adsorbed around the structure of the [EMIM]TFSI [27,28]; and (3) the [EMIM]TFSI itself has a high boiling point and is not easily volatile. After being added to the system, it changes the dependence of the hydrogel system, thereby reducing the evaporation rate of water molecules and improving the moisturizing performance of the hydrogel [29,30].

### 2.4. The Strain Sensing and Mechanism of PVA-PAA/PANi/[EMIM]TFSI Conductive Hydrogels

**Figure 5:**
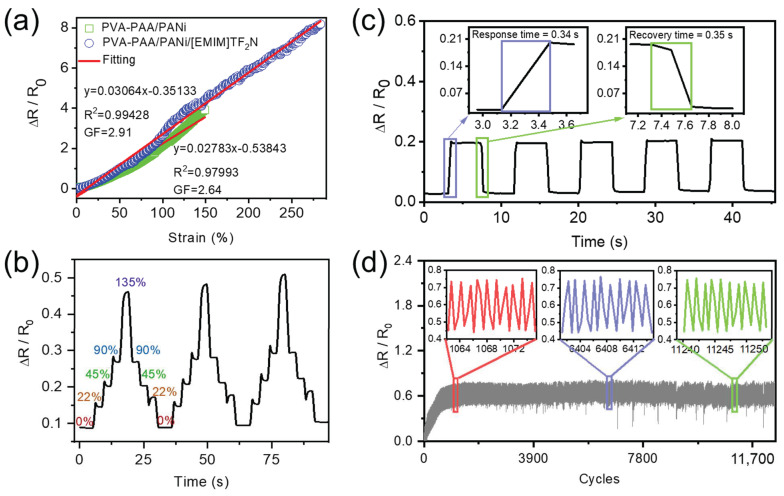
Electromechanical performances of PVA-PAA/PANi/[EMIM]TFSI strain sensor. (**a**) Relative resistance change and linear fit of the strain sensor made of PVA-PAA/PANi and PVA-PAA/PANi/[EMIM]TFSI as a function of strain. (**b**) Plot of ΔR/R_0_ vs. time with loading–unloading stretching at different strains. Incremental strains at 0, 22, 45, 90, and 135% were applied with a time frame of 5 s, and then returned to the strain 0% state in the same order. (**c**) The response time and recovery time of the PVA-PAA/PANi/[EMIM]TFSI composite sensor. (**d**) The resistance response of the PVA-PAA/PANi/[EMIM]TFSI composite sensor during 12,000 loading–unloading cycles at 100% strain under 2000 mm/s tensile speed.

As a flexible conductive material, hydrogels have shown a wide range of applications from flexible electronics and wearable devices to bioelectronic. To demonstrate their advantages in flexible and wearable devices, the conductive hydrogel was tested by cyclic tensile–release conditions. We used the optimal PVA-PAA/PANi hydrogel and PVA-PAA/PANi/[EMIM]TFSI composite hydrogel as samples, then attached them to VHB tape and connected the cables at both ends of the hydrogels to allow them to stand under ambient conditions for 24 h to obtain the strain sensors. The electromechanical performances of the PVA-PAA/PANi sensor and PVA-PAA/PANi/[EMIM]TFSI composite sensor are compared in Figure 5a, and the gauge factor (GF) of the as-prepared sensors were calculated and then the strain-resistance change rate curve of the different samples was linearly fitted. For the PVA-PAA/PANi sensor, its sensitivity GF is 2.64 at a wide linear stretching range from 0% to 150%. Meanwhile, the linear fit equation is y = 0.02783x − 0.53843 (R^2^ = 0.97993), indicating that the sample has a good linear correlation between the resistance change rate and strain. After the addition of [EMIM]TFSI, the sample’s GF value increased to 2.91, and the linear stretching range was further extended beyond 290% (the linear fit equation: y = 0.03064x − 0.35133, R^2^ = 0.99428), which is caused by the formation of a stable composite conductive network between ionic liquid and polyaniline. Those results are superior to most stretchable strain sensors from PANi-based hydrogels reported so far [16,21,34,35].

The revertive behavior of the PVA-PAA/PANi/[EMIM]TFSI composite sensor was examined by continuously stretching it at different strains. Incremental strains at 0%, 22%, 45%, 90%, and 135% were applied, and then they were returned to the strain initial state, respectively. Figure 5b shows the ΔR/R_0_ vs. time for 3 loading–unloading stretching cycles from 0% to 135% to 0% with a time frame of 5 s for each strain loading or unloading. It can be seen that resistance repeatedly changes with the stretching–relaxation cycles. In addition, the sensor has very good resilience, and the value at the time of reconciliation almost coincides with the response value, without obvious hysteresis. The sensor showed fast response time (~0.34 s) and recovery time (~0.35 s), as shown in Figure 5c. The response time and the recovery time are almost equally the same, which shows the possibility that the sensor can detect the strain response in real time and have good recovery stability. The durability of the PVA-PAA/PANi/[EMIM]TFSI composite sensor was trained by the continuous stretching–relaxation cycles (100% strain) with more than 12,000 cycles at a stretching speed 100 mm/s, exhibiting stable and reproducible resistance output (shown in Figure 5d). We compared the performance parameters of the reported PANi conductive hydrogel with our conductive hydrogel and summarized it in Table S3 [16,21,24,34,35,36,37]. In contrast, this work significantly advances PANi-based hydrogels by achieving an optimal balance of properties rarely seen in prior studies. Our PVA-PAA/PANi/[EMIM]TFSI hydrogel uniquely combines a wide linear sensing range (0–290% strain) with a high gauge factor (~2.91), fast response/recovery (0.34 s/0.35 s), and exceptional durability (>12,000 cycles). The strategic incorporation of [EMIM][TFSI] serves a triple role by drastically enhancing conductivity (0.68 S/cm), improving stretchability, and providing superior anti-drying performance (~60% mass retention after 3 days). This multi-functional design overcomes common trade-offs between sensitivity, linearity, and stability, establishing a new benchmark for robust, high-performance strain sensors in wearable electronics and soft robotics.

To further analyze the resilience and wide linear response range of our sensors, the mechanism of the PVA-PAA/PANi/[EMIM]TFSI composite sensor was discussed. In this work, we use PVA-PAA as the template framework to guide the oxidative polymerization of aniline. The continuous PANi network was closely connected with the PVA-PAA network through hydrogen bonding and electrostatic interaction. Further, the introduction of [EMIM]TFSI ionic liquids can regulate the stability of the PVA-PAA-PANi network structure and improve the conductivity of the PVA-PAA/PANi/[EMIM]TFSI composite hydrogel. The conductivity of the PVA-PAA/PANi/[EMIM]TFSI composite hydrogel depended on the formation of a continuous conductive network of PANi and the three-dimensional (3D) cross-conductive network formed between PANi and [EMIM]TFSI ionic liquid. With the small stretch, due to the action of transverse Poisson compression during the stretching process, slippage between the PANi and PVA-PAA network happens, which causes part of the PANi conductive network to be deformed and elongated [38,39], resulting in an increase in resistance. However, due to the formation of the near-threshold PANi continuous conductive network in the PVA-PAA/PANi/[EMIM]TFSI conductive hydrogel [40], the conductive hydrogel still maintained a stable resistance response under low strain. On the other hand, as the strain gradually increased, the PANi conductive network gradually deformed and elongated without degradation. However, due to the good fluidity and excellent electron-ion transport properties of [EMIM]TFSI, [EMIM]TFSI connected the surrounding PANi conductive network [29], making a stable sensing pathway in the conductive hydrogel. It was precisely because [EMIM]TFSI can form an electron-ion conductive channel between the PVA-PAA-PANi connected network that the PVA-PAA/PANi/[EMIM]TFSI composite hydrogel maintained an effective resistance response in a large strain range [41,42,43]. At this time, the special [EMIM]TFSI-connected PANi conductive network constituted the main electronic channel.

**Figure 6:**
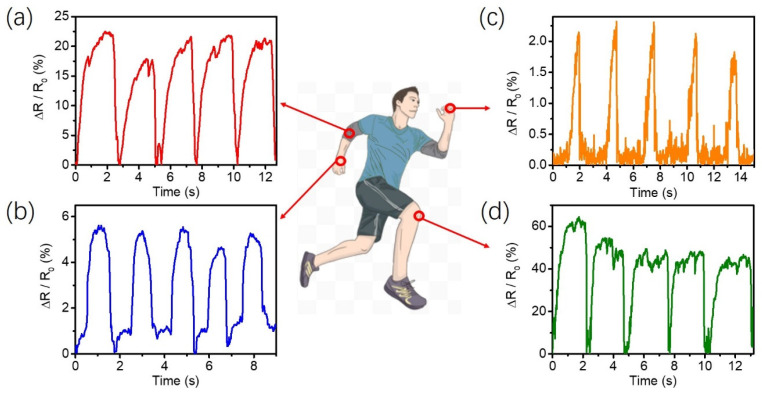
The PVA-PAA/PANi/[EMIM]TFSI conductive hydrogel was adhered to the fingers, wrists, elbows, and knees to monitor the physiological signals of limb movement of (**a**) elbow, (**b**) wrist, (**c**) finger, and (**d**) knee bending activities.

Good moisturizing ability, excellent strain-sensing linearity, and repeatable sensing capabilities show the PVA-PAA/PANi/[EMIM]TFSI conductive hydrogel to be an effective an on-skin wearable sensor to monitor various, limited human activities. As shown in Figure 6, when the hydrogel sensor was installed at the joint, the hydrogel could be stretched and bended at a certain angle to produce a repeatable resistance change rate. Firstly, the hydrogel sensor was installed on the finger (Figure 6a). When the finger was bent to 45° for multiple cycles, the hydrogel showed a stable and steady resistance change rate. Similarly, when hydrogel sensors were installed on wrists (Figure 6b), elbows (Figure 6c), and knees (Figure 6d) to monitor body signals, the hydrogel sensor exhibited a stable and repeatable resistance change rate. In addition, the human joint bending monitoring data (such as fingers, wrists, elbows, and knees) can be used for the correction and treatment of special crowd (joint patients, athletes) activities and sports postures. These encouraging results indicated that the PVA-PAA/PANi/[EMIM]TFSI conductive hydrogel has great potential in flexible wearable devices.

## 3. Conclusions

In summary, we have successfully developed a high-performance, linearly responsive, and stretchable strain sensor based on a novel multiple-network conductive hydrogel composed of PVA, PAA, in situ polymerized PANi, and the ionic liquid [EMIM][TFSI]. This composite hydrogel overcomes several key limitations of conventional conductive hydrogels, including poor dehydration resistance, signal hysteresis, and nonlinear response. The incorporation of [EMIM][TFSI] played a multifaceted role as a plasticizer, anti-drying agent, and conductivity enhancer, leading to a remarkable 17-fold increase in electrical conductivity and a significant improvement in stretchability. The optimized hydrogel exhibits an exceptional combination of properties: high electrical conductivity (0.68 S/cm), a wide linear sensing range (0–290% strain), a high gauge factor (2.91), fast response/recovery times (0.34 s/0.35 s), excellent mechanical strength (3.7 MPa tensile strength, ~1.3 MPa modulus), and outstanding cyclic durability over 12,000 stretching–relaxation cycles. Furthermore, the hydrogel demonstrates markedly improved anti-drying performance, retaining approximately 60% of its initial mass after 3 days at room temperature. The synergistic interactions between the PVA-PAA network and PANi, facilitated by hydrogen bonding and electrostatic forces, along with the formation of a stable PANi-[EMIM][TFSI] conductive network, are identified as the key factors enabling this comprehensive performance. This work provides a robust and scalable material strategy for developing reliable strain sensors, showcasing great potential for applications in wearable electronics, soft robotics, and advanced human–machine interfaces.

## 4. Materials and Methods

### 4.1. Preparation of PVA-PAA/PANi/[EMIM]TFSI Conductive Hydrogels

Polyacrylic acid (PAA, Mw = 3000, 30 wt.% in water) was obtained from Damao Chemical Reagent Factory in Tianjin, China. Glutaraldehyde (GA, 25 wt.% in water) was sourced from Xilong Scientific in Shantou, China. [EMIM]TFSI ionic liquid was supplied by Chengjie Chem. Co. in Shanghai, China. Poly(vinyl alcohol) (PVA), ammonium persulfate (APS), sulfuric acid (H_2_SO_4_), and aniline (ANi, monomer) were purchased from Aladdin Chemistry in Shanghai, China. All the reagents were used as received.

Firstly, we weighed 7.00 g of water and added 0.412 mL of H_2_SO_4_ to prepare a 1.0 M H_2_SO_4_ solution. Next, 0.1 mL of [EMIM]TFSI ionic liquid (IL) was added, followed by 0.5 g of PVA after stirring for 10 min. The mixture was then stirred and dissolved at 95 °C. Once the PVA was fully dissolved, it was cooled to room temperature. Subsequently, 2.5 g of PAA and 0.2 g of aniline were added, then stirred for 30 min to form a stable and uniform aqueous polymer solution, which was then transferred to an ice–water bath. Next, 1.25 g of initiator APS aqueous solution (20 wt.%) was added to polymerize the aniline monomer. The mixture was kept in the ice–water bath for 2 h, then at room temperature (RT) for 12 h with continuous stirring throughout. After removing the bubbles, 75 μL of glutaraldehyde was added to the polymer mixture and stirred for 30 s. The mixture was then poured into an acrylic template (60 mm × 60 mm × 2 mm) and cross-linked at 30 °C for 24 h to form the PVA-PAA/PANi/[EMIM]TFSI conductive hydrogels. Different types of conductive hydrogels can be prepared by varying parameters such as PVA:PAA ratio, [EMIM]TFSI content, and cross-linker amount. The detailed variations in preparation methods used to achieve different PVA-PAA/PANi/[EMIM]TFSI conductive hydrogels are summarized in Table S2.

### 4.2. Fabrication of the Strain Sensor Based on PVA-PAA/PANi/[EMIM]TFSI Hydrogels

The different PVA-PAA/PANi polymer mixtures with the cross-linking agent were first poured into a polytetrafluoroethylene template (50 mm × 20 mm × 1 mm) and cross-linked at RT for 24 h to form conductive hydrogels. Subsequently, a commercial very high bonding tape (VHB, 3M Corp., Saint Paul, MN, USA) was used as the adhesive substrate. Cables were attached at both ends of the conductive hydrogel and then attached onto the VHB tape with 24 h of stabilization to fabricate the strain sensors.

## Data Availability

The raw data supporting the conclusions of this article will be made available by the authors on request.

## Supplementary Materials

The following supporting information can be downloaded at: https://www.mdpi.com/article/10.3390/gels11120966/s1, Figure S1: The schematic flowchart of the experimental results and multiple detection methods involved in this work. Figure S2: The freeze-dried PVA-PAA/PANi conductive hydrogels can almost restore the hydrogel to its original height after suffering from compression-recovery. Figure S3: The conductivity of different hydrogels with and without H_2_SO_4_. Figure S4: The equivalent circuit models fitted from the electrochemical impedance spectra of the PVA-PAA/[EMIM]TFSI and PVA-PAA/PANi/[EMIM]TFSI hydrogel. Figure S5: The Raman spectroscopy of the PVA-PAA/PANi/[EMIM]TFSI conductive hydrogel. Table S1: The fitted electrical parameters derived from the equivalent circuit models. Table S2: The formula table for preparing PVA-PAA/PANi/[EMIM]TFSI conductive hydrogels. Table S3: Performance comparison table on PANi-based conductive hydrogel.
