# Supplementary material for: Linearly Responsive, Reliable, and Stretchable Strain Sensors Based on Polyaniline Composite Hydrogels
Source: Gels. 2025 Nov 29;11(12):966. doi: 10.3390/gels11120966 (PMC12732904; doi:10.3390/gels11120966)
Supplement: Supplementary file 1 [file gels-11-00966-s001.zip › gels-3962006-supplementary.pdf]

Supporting Information

Linearly Responsive, Reliable, and Stretchable Strain Sensors Based on Polyaniline Composite Hydrogels

*Chubin He, Xiuru Xu**

1. Material Characterization

1.1 Physical Characterization

The chemical structures of different hydrogel samples were characterized by Fourier transform infrared spectrometer with the diffuse reflection model (Spectrum One Version B, PerkinElmer Inc. Waltham, USA) and Raman Spectroscopy (Lab RAM Xplora, Horiba Jobin Yvon S.A.S, France). The test wavelength was from 500 to 4000 cm^{-1}. Scanning Electron Microscopy (JSM-6380LV, JEOL, Japan) provide visual evidence of the dispersion of the PANi hierarchical structure within the PVA-PAA matrix. BET test (BSD-660M, BeiShiDe Instrument, China) on hydrogel samples to obtain the pore size distribution. Thermogravimetric analysis (TG 209 F3 Nevio, Netzsch, Germany) was conducted to quantify the anti-drying mechanism. The test temperature range was from 25 to 250 °C. The electrochemical impedance analysis (Chi660e, CH Instruments, Shanghai China) was performed on different gel samples. All measurements were conducted at the open-circuit potential with the testing frequency from 10 mHz to 100 kHz.

1.2 Mechanical Tests

In order to measure the mechanical properties of the hydrogel, a universal stretching machine (E1000, Instron, Boston, USA) was used to perform a tensile test on the hydrogel at room temperature. Cut all hydrogel samples into splines with 17 mm length, 6 mm width, and 0.2 mm thickness, respectively. During the test, the stretching speed was set to 100 mm/min. At the same time, five samples needed to be measured for each component, and the results

were averaged. The elastic modulus was calculated from the slope of the linear region of the stress-strain curve (ε = 5-20 %).

1.3 Electrical Conductivity and Strain-Sensing Performance

The resistance values of all hydrogels were tested by using a digital multimeter (34465A, Keysight, Guangzhou, China) and calculated the conductivity the conductivity of the samples by **Equation 1**: $\sigma = \frac{1}{\rho} = \frac{L}{R \cdot S}$, where σ was electrical conductivity, ρ was resistivity, L was the length of samples, S was the surface area of samples. Universal stretching machine and digital multimeter (34465A, Keysight, Guangzhou, China) were used to test the sensor performance of hydrogels. Firstly, the hydrogels were fixed in a universal stretching machine. At the same time, the samples were connected to the digital multimeter, which can record the change curve of the resistance of the hydrogels with strain.

1.4 Drying Resistance Characterization

The prepared conductive hydrogels were stored at room temperature for 6 days, and their anti-drying performance was evaluated by monitoring the remaining mass and relative resistance change of the hydrogel samples during the testing period. Each set of experiments was performed in triplicate. For the remaining mass: the initial mass (W_0) of the as-prepared hydrogel was first recorded. The hydrogel was then stored under ambient room temperature conditions, and its mass (W_t) was measured again after various time intervals. The mass retention was calculated using the following formula: Mass Retention (%) = (W_t / W_0) × 100%. For the relative resistance change, the initial resistance (R_0) of the freshly prepared conductive hydrogel was first measured. The resistance (R_t) of the sample was then measured after different storage times. The relative resistance change after various storage periods was calculated using the formula: (R_t - R_0) / R_0.

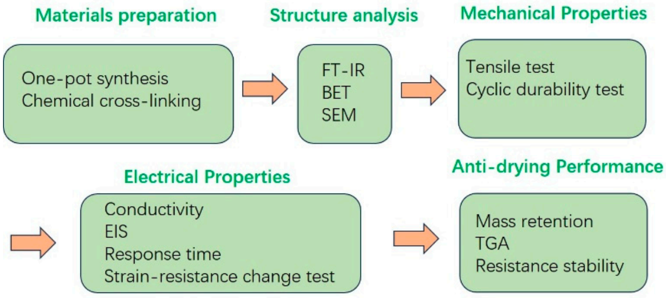

Figure S1. The schematic flowchart of the experimental results and multiple detection methods involved in this work.

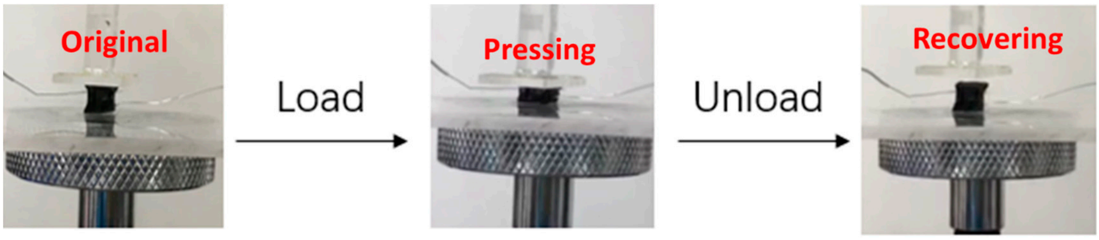

Figure S2. The freeze-dried PVA-PAA/PANi conductive hydrogels can almost restore to its original height after suffered from compression-recovery.

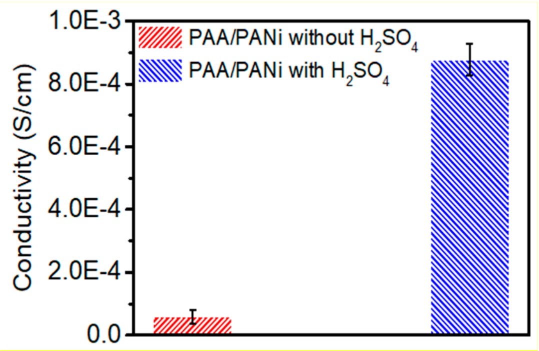

Figure S3. The conductivity of different samples. Compared with the PAA-PANi sample without H_2SO_4, the electrical conductivity of the PAA-PANI-H_2SO_4 sample is effectively increased because the doping of the PANi chain by the sulfate ion increases the electron separation of the PANi chain.

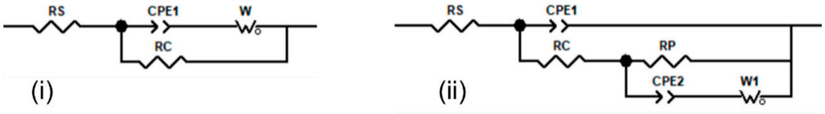

Figure S4. The equivalent circuit models fitted from the electrochemical impedance spectra of the hydrogel samples: (i) PVA-PAA/ [EMIM]TFSI, (ii) PVA-PAA/PANi/[EMIM]TFSI.

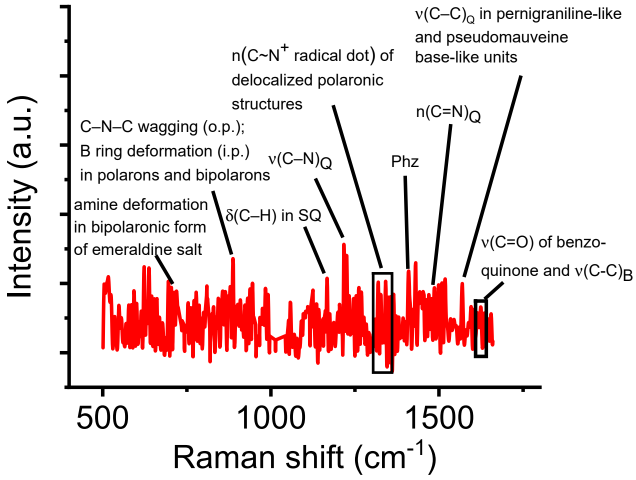

Figure S5. The Raman spectroscopy of the PVA-PAA/PANi/[EMIM]TFSI conductive hydrogel.

Table S1. The fitted electrical parameters derived from the equivalent circuit models

	PVA-PAA/ [EMIM]TFSI	PVA-PAA/PANi/[EMIM]TFSI
R_s ($\Omega\cdot cm^2$)	420.6	295.4
R_c ($\Omega\cdot cm^2$)	534430	176.3
CPE_1-T ($\mu F\cdot cm^{-2}$)	0.0023	3.2277 * 10^{-5}
n_1	0.2398	0.4171
R_P ($\Omega\cdot cm^2$)	-	1.4086 * 10^7
CPE_2-T ($\mu F\cdot cm^{-2}$)	-	0.0002512
n_2	-	0.9132
W_R ($\Omega\cdot cm^2$)	243.7	15157
W_T ($\mu F\cdot cm^{-2}$)	0.0571	232.1
W_P	0.4573	0.5519

Table S2. The detailed variation in preparation crafts applied to achieve different PVA-PAA/PANi/[EMIM]TFSI conductive hydrogels

	H_2O (g)	H_2SO_4 (mL)	ANi (g)	APS solution		PVA (g)	PAA (g)	[EMIM]TFSI (μL)	GA (μL)
				APS (g)	H_2O (g)				
1	7	0.412	0.2	0.25	1	1	2	-	75
2	7	0.412	0.2	0.25	1	0.75	2.25	-	75
3	7	0.412	0.2	0.25	1	0.5	2.5	-	75
4	7	0.412	0.2	0.25	1	0.375	2.625	-	75
5	7	0.412	0.2	0.25	1	0.3	2.7	-	75
6	7	0.412	0.2	0.25	1	0.5	2.5	-	25
7	7	0.412	0.2	0.25	1	0.5	2.5	-	50
8	7	0.412	0.2	0.25	1	0.5	2.5	-	60
9	7	0.412	0.2	0.25	1	0.5	2.5	-	75
10	7	0.412	0.2	0.25	1	0.5	2.5	-	100
11	7	0.412	0.2	0.25	1	0.5	2.5	25	75
12	7	0.412	0.2	0.25	1	0.5	2.5	50	75
13	7	0.412	0.2	0.25	1	0.5	2.5	75	75
14	7	0.412	0.2	0.25	1	0.5	2.5	100	75
15	7	0.412	0.2	0.25	1	0.5	2.5	300	75

H_2SO_4: sulfuric acid; ANi: aniline; APS: ammonium persulfate; PVA: poly(vinyl alcohol); PAA: polyacrylic acid; GA: glutaraldehyde; [EMIM]TFSI: 1-ethyl-3-methylimidazolium bis[(trifluoromethyl)sulfonyl]imide.

Table S3. Performance comparison table on PANi-based conductive hydrogel

	Conductivity (S/cm)	Fracture strain (%)	Linear sensing range (%)	Responsibility	Strain Sensitivity GF
PANi/PSS-UPy/Fe^{3+} [1]	14.2	300	0-300	Hysteresis 30s	3.4
PVA/PANi/glycerin [2]	0.335	472	——	small hysteresis circle	2.14
UHMWPE/PANi [3]	0.87	10	0-10	——	15.47
PANi/PAAMPSA/PA [4]	2	1900	——	Good	14
PANi/PAA/PA [5]	0.12	500	0-100，100-500	Good flexibility, but obvious hysteresis	11.6（0-100）；4.7（100-500）
PAA/PANi/PA/ Pluronic F127 diacrylate [6]	5.12	1160	0-800，800-1130	obvious hysteresis	0.6（0-800）1.05（800-1130）
PAAm/PANi/chitosan microspheres [7]	0.05	>600	——	——	——
Our work*	0.675	290	0-290	no obvious lag	2.5

PANi: polyaniline; PAAm: polyacrylamide; PVA: poly(vinyl alcohol); PAA: polyacrylic acid; PA: phytic acid; PAAMPSA: poly(2-acrylamido-2-methyl-1-propanesulfonic acid); PSS: poly(4-styrenesulfonate); UPy: 2-ureido-4[1H]-pyrimidinone; UHMWPE: UHMWPE filament yarn (444dtex/406f).

References

1. Chen, J.; Peng, Q.; Thundat, T.; Zeng, H. Stretchable, injectable, and self-healing conductive hydrogel enabled by multiple hydrogen bonding toward wearable electronics. Chemical Materials 2019, 31, 4553-4563, https://doi.org/10.1021/acs.chemmater.9b01239.
2. Hu, C.; Zhang, Y.; Wang, X.; Xing, L.; Shi, L.; Ran, R. Stable, strain-sensitive conductive Hydrogel with antifreezing capability, remoldability, and reusability. ACS Applied Materials & Interfaces 2018, 10, 44000-44010, https://doi.org/10.1021/acsami.8b15287.
3. Hong, J.; Pan, Z.; ZheWang; Yao, M.; Chen, J.; Zhang, Y. A large-strain weft-knitted sensor fabricated by conductive UHMWPE/PANI composite yarns. Sensors and Actuators A: Physical 2016, 238, 307-316, https://doi.org/10.1016/j.sna.2015.12.028.
4. Lu, Y.; Liu, Z.; Yan, H.; Peng, Q.; Wang, R.; Barkey, M.E.; Jeon, J.W.; Wujcik, E.K. Ultrastretchable conductive polymer complex as a strain sensor with a repeatable autonomous self-healing ability. ACS Applied Materials&Interfaces 2019, 11, 20453-20464, https://doi.org/10.1021/acsami.9b05464.
5. Wang, T.; Zhang, Y.; Liu, Q.; Cheng, W.; Wang, X.; Pan, L.; Xu, B.; Xu, H. A self-healable, highly stretchable, and solution processable conductive polymer composite for ultrasensitive strain and pressure sensing. Advanced Functional Materials 2018, 28, 1705551, doi:10.1002/adfm.201705551.
6. Wang, Z.; Zhou, H.; Lai, J.; Yan, B.; Liu, H.; Jin, X.; Ma, A.; Zhang, G.; Zhao, W.; Chen, W. Extremely stretchable and electrically conductive hydrogels with dually synergistic networks for wearable strain sensors. Journal of Materials Chemistry C 2018, 6, 9200-9207, https://doi.org/10.1039/C8TC02505C.
7. Duan, J.; Liang, X.; Guo, J.; Zhu, K.; Zhang, L. Ultra-stretchable and force-sensitive hydrogels reinforced with chitosan microspheres embedded in polymer networks. Advanced Materials 2016, 28, 8037-8044, https://doi.org/10.1002/adma.201602126.
